# Coronary artery bypass grafting and sensorineural hearing loss, a cohort study

**DOI:** 10.1186/1472-6815-5-12

**Published:** 2005-12-10

**Authors:** Omer Ashraf

**Affiliations:** 1 Aga Khan University, Stadium Road, Karachi, 74800, Pakistan

## Abstract

**Background:**

Sudden sensorineural hearing loss is routinely encountered by the otologist. The etiology is varied and often identifiable. One of the relatively less frequent causes is surgery. Apart from being an established entity with otological surgeries, sensorineural hearing loss has also been known to occur after non-otological procedures under general anesthesia. Commonest amongst these procedures is cardiopulmonary bypass, an association that has long been recognized. However, despite the proposition of diverse hypotheses in the past, the pathophysiology remains unclear.

**Methods:**

The study is a prospective matched cohort study that will be carried out in Aga Khan University Hospital, Karachi, Pakistan. Participants among exposed would include all those patients who would be undergoing coronary artery bypass surgery in the hospital who fall under the criteria for inclusion. Unexposed group would comprise of patients undergoing a non-bypass procedure of similar duration under the same type of anesthesia who meet the selection criteria. Both these groups will undergo audiometric testing at our hospital on three different occasions during the course of this study. Initially before the procedure to test the baseline hearing capacity; then one week after the procedure to assess any changes in hearing ability following the surgery; and finally a third audiogram at six weeks follow-up to assess further changes in any hearing deficits noted during the second phase of testing. Certain variables including the subjects' demographics and those concerning the procedure itself will be noted and used later for risk factors analysis. A detailed past medical and surgical history will also be obtained. Data analysis would include calculation of relative risk and significance of the results, by running the chi-square test. Other statistical tests like Fisher exact test may then be employed to facilitate data interpretation. Continuous scale may then be employed and multivariate linear regression used.

**Discussion:**

This study is planned to obtain a better understanding of the correlation between sudden sensorineural hearing loss and cardiopulmonary bypass. Being the first major cohort trial in this line of investigation, the project is designed to identify the existence of any significant relationship between cardiopulmonary bypass and sensorineural hearing deficit.

## Background

Hearing is one of the most significant of human senses. For an individual it is perhaps the most crucial link in communication with the outside world. It's a prerequisite for a fulfilling life and career. Any deficit in it is as frightening for the person as a loss of vision. It's a handicap potentially severe enough to deny a person a normal life and livelihood.

Sensorineural hearing loss (SNHL) is often encountered by the ENT surgeon. It is established globally as a significant morbidity. Even more traumatizing is the development of a sudden sensorineural hearing loss by a patient, a disease afflicting 1 in every 5000 patients in the general population [1]. Various etiologies are proposed to explain the sudden SNHL in general population and include inflammatory (bacterial, viral and autoimmune), vascular (hemorrhage, microemboli, thrombosis), metabolic (hormonal, chemical, drugs), traumatic (direct, noise, surgical), membrane rupture, functional, or idiopathic. In many of the unidentifiable cases there is still debate between a viral versus vascular etiology.

Sensorineural hearing loss can also be explained as a surgical complication of otologic surgeries. However, what has raised the great interest of medical professionals around the world in recent decades is the occasional development of sudden sensorineural hearing loss with non-otologic surgeries carried out under general anesthesia. A considerable majority of these surgeries include cardiopulmonary bypass procedures. The first report of this kind was made by Arenberg (1972) [2] who reported sudden unilateral deafness immediately following cardiopulmonary bypass. Earlier Brownson et al (1971) [3] had tried unsuccessfully to achieve the same in a prospective study with a limited sample. This was followed by a larger retrospective study by Plasse et al [4,5] in which he evaluated 7000 patients and found the incidence of SNHL with aortocoronary bypass surgery to be 1 in 1000 (0.1%). However, these results are highly questionable considering the presence of false-negatives and false-positives associated with retrospective studies. It has been established since that SNHL can be objectively determined only in routine and prospective evaluation.

There was controversy in the past in the demarking of criteria for SNHL. This was evident in the study by Shapiro et al [6] who applied overly inclusive criteria and found the hearing loss to be 13.2% in a prospective study involving 68 patients. Doubts were always raised though, regarding the inclusion in his study of the patients who reported a hearing deficit of up to 10 dB, which can be owing to the potential variations and audiometric testing errors due to patient concentration and co-operation during the testing.

This was followed by two case reports from Millen et al [7] and a prospective study by Ness et al [8].

A review of the literature indicates several distinct variants. First, it is found that the SNHL in patients undergoing coronary artery bypass grafting (CABG) occurs in an older age group with known preexisting cardiovascular disease [1]. Second, there is a clear male to female preponderance in the incidence of these cases that cannot merely be explained by the greater number of male patients undergoing the procedure [4].

It has also been found that there is little or no recovery from the insult with a partial recovery of hearing occurring in less than 50% of patients [1]. Another point is the absence of vertigo in most of these cases [4,5]. Tinnitus may also be found [6].

The magnitude of the problem has long been recognized considering the incidence rates that are reported up to 10–15% [6], and the fact that even in 1980 over 100,000 bypass operations were carried out in the United States [9]. Various etiologies had thus been proposed to look into the pathophysiology of this phenomenon and thus offer a possible preventive or curative measure. Microembolic phenomena (fat, air or particulate thrombi)[2], perioperative hypotension or perfusion failure [6], hypercoagulable states [10], and ototoxic drug usage [6] are some of the better recognized ones.

However, many of the mechanisms involving CABG (e.g. fat emboli, anti-foaming agents) do not apply to those other non-otologic surgeries, which have reported sudden idiopathic SNHL. Since all of the reported cases have occurred under general or spinal anesthesia, the hemodynamic fluctuations during induction and maintenance of anesthesia common to both of these could be a factor [11]. Cochlear membrane leaks and perilymphatic fistulae have been invoked as possible mechanisms for loss of hearing. Both implosive and explosive mechanisms have been implicated [12]. Nitrous oxide is often blamed as it can generate very high pressures in the middle ear [13,14]. However, there have been described cases in which nitrous oxide was not used, and its presence in many cases may only reflect its very widespread anesthetic use. It is also important to exclude a brainstem event causing bilateral hearing loss [15,16], though in these reports other neurological signs like ataxia, dysarthria, gaze palsies and multiple cranial nerve and neurological deficits have been mentioned alongside.

## Methods

### Study design

This is a prospective matched cohort study.

### Setting

Aga Khan University Hospital, Karachi, Pakistan.

### Inclusion criteria for exposed group

All patients undergoing CABG who give consent and can comply with the testing. They will be taken by consecutive sampling.

### Inclusion criteria for unexposed group

All patients who qualify for the criteria of being age and sex matched with the exposed (on an individual basis) undergoing a non-cardiac and non-otologic surgery under general anaesthesia, of similar time duration, who give consent and comply with the testing. There is no report in literature of association of SNHL with any specific non-otologic, non-cardiac surgery, apart from certain neurosurgical procedures, especially around the brainstem and/or the VII-VIIIth cranial nerve complex. So any surgery apart from the above mentioned, which employs the same type of anaesthesia and is of similar time duration (the two pertinent factors) can be taken as control to generate the sample. We have decided to use the patients undergoing surgeries for head and neck tumors, not having any otologic, brain stem or the VII-VIIIth cranial nerve complex involvement, as members of the unexposed group. The patient age group is likely to be similar and the procedure will last a similar time period. They are being taken on a basis of one unexposed per exposed. Age, within a five-year period, and sex matching will be done, for the selection of unexposed.

### Exclusion criteria

1. Those who don't give consent.

2. Those who can't comply with the testing.

3. Those who have a previous history of ear surgery.

### Sample size calculation

As regards sample size calculation, there are two widely varying outcomes of hearing loss, in literature, 0.1% and 13.2%. Both studies had their intrinsic flaws, the former being a retrospective study, and the latter using overly inclusive criteria. Taking the highest reported incidence of deafness to be 13.2 % with a 95% level of significance, 80% power, the sample size comes out to be 138 (69 each group). The expected frequency of disease in the unexposed group is taken as equivalent of that in the general population which is 0.2%.

However, to further increase the power of the study, the best possible approach (that is expected to generate a manageable sample size) is to take the frequency of disease as 6.55% (between the outcomes of the two above mentioned studies), then the sample size, with a 95% level of significance and 80% power, is 290 (145 per group), which is manageable. This estimate is both manageable and appropriate considering the intrinsic errors in both the quoted studies.

### Data collection tool

Three audiograms will be done for objective assessment of patients and one questionnaire will be filled from interviewing of the patients, for subjective analyses. Certain perioperative data will be noted.

The first audiogram will be done pre-operatively to assess the baseline level of a subject's hearing. The second audiogram will be done after the procedure, after one week, prior to the discharge of patients. This will assess the alteration in hearing, if any, of the subject. The third and final audiogram will be done at six weeks follow up. This will aid in providing information about any late deterioration or recovery.

The audiogram will assess auditory function of both ears obtained via a complete audiometric evaluation in a soundproof room in the ENT clinic of our hospital, including pure-tone air and bone conduction levels, speech reception thresholds, and speech discrimination score testing.

The questionnaire will make note of patient demographics and various questions that comprise an otologic and past medical and surgical history asked to determine if the patient had any prior hearing loss, ear disease, ear surgery, known ototoxic medication usage, tinnitus, vertigo, family history of hearing loss, noise exposure, neurological disease, diabetes, hyperlipidemia, hypertension and hypotension. After the procedure it will be inquired if the patient had the subjective complaint of hearing loss, tinnitus, vertigo, imbalance, any ear pain or discharge. Similar questions will be asked at the six week follow up.

Perioperative data collected on each patient will include the type of surgery (CABG simply or CABG with some other surgery like valve replacement), time on perfusion pump, the type of pump used, the type of filter used, aortic cross clamp time, blood loss, fluids, maximum and minimum systolic and diastolic pressures during surgery, pulse, oxygen saturation, any straining, emesis or increased venous pressure from over ventilation, extension of neck for long period during the procedure, postoperative complications (e.g. arrhythmias, prolonged hypotension, hypertension and neurological changes), and medication usage.

Also noted will be the components of anesthesia, vitals, blood loss, fluids and medications in the recovery room. Any other medical ailment or surgical procedure during the six-week post-operative period will also be noted in detail.

### Data analysis

Data analysis will be carried out by entering the data into the Microsoft Windows based Statistical Package for the Social Sciences (SPSS- released 10.1, standard version, copyright SPSS; 1989–1999). Anonymity of the subjects will be maintained. Relative risk will be calculated. Significance of the results will be obtained by running the chi-square test and later further interpretations carried out by using the Fisher exact test. Change in hearing acuity may be calculated on a continuous scale (as by calculating the mean hearing change in the two groups, and comparing them between the exposed and the unexposed group). Data analysis will then be carried out, in case of usage of continuous variables, by multivariate linear regression.

## Discussion

What is the exact cause, what then is the pathophysiology and how can we apply better curative and preventive measures to eliminate any further incidence of the sudden SNHL from not just CABG procedures but other non-otologic procedures as well, is a source of some concern to surgeons around the planet and the solution lies in evidence based medicine in this direction. The rationale of this study is not just the need for better understanding of the issue but also to reduce the psychological trauma and morbidity encountered by patients, and to help in increasing patient confidence in life-saving procedures as CABG.

## Abbreviations

Sensorineural hearing loss: SNHL

Ear, nose and throat: ENT

Coronary artery bypass grafting: CABG

## Competing interests

The author(s) declare that they have no competing interests.

## Authors' contributions

Being the sole author, Omer Ashraf was involved in design of study, drafting and revision of the article and in final approval of the manuscript version to be published.

## Pre-publication history

The pre-publication history for this paper can be accessed here:


